# OP70 Implementation Of An Online Consultation Hub To Facilitate Consumer Engagement In Health Technology Assessment Processes

**DOI:** 10.1017/S0266462324001296

**Published:** 2025-01-07

**Authors:** Rebecca Trowman, Jo Watson, Bella Beach-Mills

## Abstract

**Introduction:**

In 2021, the Australian Government Department of Health and Aged Care’s Consumer Evidence and Engagement Unit (CEEU) launched an online consultation hub to provide a centralized pathway for consumer engagement in health technology assessment (HTA) processes. The hub enables consumers (patients, carers, health professionals, and citizens) to provide commentary on items being considered by HTA committees for subsidization.

**Methods:**

A survey was developed by the CEEU, committee members, and consumer representatives to facilitate consultation on applications assessed by the Pharmaceutical Benefits Advisory Committee (PBAC)—a principal Australian HTA committee. Questions were designed as simple and engaging, including guidance on the type of information required. Responses are summarized thematically by efficacy, safety, accessibility, and quality-of-life impacts of the proposed health technology. New surveys are launched to coincide with each PBAC meeting agenda publication and allow a ten-week consultation period. Awareness of consultations is supported by the CEEU’s HTA Engage e-newsletter, which alerts the public and targeted stakeholders.

**Results:**

The hub surveys have enabled streamlined consumer commentary to be provided for PBAC considerations. It has also allowed increased time for quality consultation with stakeholders. The success of the hub is further demonstrated in the current development of a similar survey for another principal committee, the Medical Services Advisory Committee (MSAC), to transition its consultation processes to the hub. Of note, while consumers’ feedback on the hub is positive, there remains a desire for educational resources and face-to-face interactions to support consumer engagement in HTA processes. The CEEU are developing materials to address and further support this need.

**Conclusions:**

In a time when technological communication can be optimized to complement face-to-face conversations, it is vital consumer engagement in HTA processes follows suit. To facilitate continued engagement that is sustainable for both present and future Australians, the CEEU continues to evolve a strategy regarding virtual consultations to increase consumer awareness and education and promote effective participation in Australian HTA.

